# Self-Assembly of N-Rich Triimidazoles on Ag(111): Mixing the Pleasures and Pains of Epitaxy and Strain

**DOI:** 10.1021/acs.jpcc.3c03325

**Published:** 2023-11-17

**Authors:** Aisha Ahsan, Xing Wang, Rejaul Sk, Mehdi Heydari, Luiza Buimaga-Iarinca, Christian Wäckerlin, Elena Lucenti, Silvio Decurtins, Elena Cariati, Thomas A. Jung, Ulrich Aschauer, Shi-Xia Liu

**Affiliations:** ∇ Laboratory for X-ray Nanoscience and Technologies, Paul Scherrer Institute, Villigen-PSI 5232, Switzerland; † Department of Physics, University of Basel, Klingelbergstrasse 82, Basel 4056, Switzerland; ‡ Department of Chemistry, Biochemistry and Pharmaceutical Sciences, University of Bern, Freiestrasse 3, Bern 3012, Switzerland; ○ National Institute for Research and Development of Isotopic and Molecular Technologies (INCDTIM), Donat Str., Cluj-Napoca 67-103, Romania; ⊥ Institute of Physics, École Polytechnique Fédérale de Lausanne Station 3, Lausanne 1015, Switzerland; ∥ Institute of Chemical Sciences and Technologies “Giulio Natta” (SCITEC) of CNR, via Golgi 19, Milano 20133, Italy; ¤ Department of Chemistry and Physics of Materials, University of Salzburg, Jakob-Haringer-Str. 2A, Salzburg 5020, Austria; § Department of Chemistry, Università degli Studi di Milano and INSTM RU Via Golgi 19, Milano 20133, Italy

## Abstract

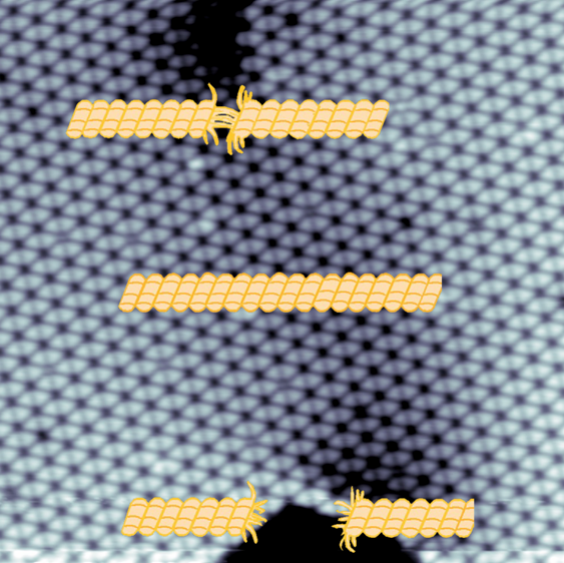

In the present report, homochiral hydrogen-bonded assemblies of heavily N-doped (C_9_H_6_N_6_) heterocyclic triimidazole (TT) molecules on an Ag(111) substrate were investigated using scanning tunneling microscopy (STM) and low energy electron diffraction (LEED) techniques. The planar and prochiral TT molecules, which exhibit a threefold rotation symmetry and lack mirror symmetry when assembled on the substrate, carry multiple hydrogen-bonding donor and acceptor functionalities, inevitably leading to the formation of hexameric two-dimensionally extended assemblies that can be either homo- (*RR*/*SS*) or heterochiral (*RS*). Experimental STM data showing well-ordered homochiral domains and experimental LEED data are consistent with simulations assuming the *R*19.1° overlayer on the Ag(111) lattice. Importantly, we report the unexpected coincidence of spontaneous resolution with the condensation of neighboring islands in adjacent “Janus pairs”. The islands are connected by a characteristic fault zone, an observation that we discuss in the context of the fairly symmetric molecule and its propensity to compromise and benefit from interisland bonding at the expense of lattice mismatches and strain in the defect zone. We relate this to the close to triangular shape and the substantial but weak bonding scheme beyond van der Waals (vdW) of the TT molecules, which is due to the three N-containing five-membered imidazole rings. Density functional theory (DFT) calculations show clear energetic differences between homochiral and heterochiral pairwise interactions, clearly supporting the experimental results.

## 1 Introduction

The symmetry of molecules and its spatial matching with point lattices determine the formation of crystals. Likewise, at surfaces and interfaces, it is the degree of lattice mismatch and commensurability that controls nucleation leading to epitaxial, fractal, or granular structures in the various growth modes. In terms of point symmetry or chirality, the phenomenon of spontaneous resolution^1−3^ is highly desirable for the separation of racemates^4^ but remains difficult to predict or control in the context of specific compounds that must be separated into two enantiomeric components after synthesis. To promote enantiomer separation, considerable efforts have been made to exploit the effect of host–guest interactions, for example, in metal organic frameworks (MOFs), or to template them at chiral sites in MOFs or on surfaces.^5−8^ The key to all these effects is the degree of symmetry matching of molecules with a potential substrate, and only in a few cases does spontaneous racemic separation (resolution) occur. Whether or not spontaneous separation occurs depends on the critical balance of intermolecular bonding, registry effects, and shape. It is also important to recall that the interaction/binding capabilities of each individual molecule are key here^9,10^: For an unfunctionalized molecule, chiral separation is limited to van der Waals condensation, i.e., molecular shape recognition.^11−15^

If more specific hydrogen, ionic, or coordination bonds^16^ can be formed with the molecules at the recognition site, the recognition and separation of enantiomers become more likely.

In the following, we have chosen cyclic triimidazole molecules with their shape deviating from hexagonal symmetry due to the three N-containing five-membered rings (imidazole rings) linked to a central triazinic core, quite in contrast to the compounds that strictly follow a sixfold symmetric pattern. The molecule is thus prochiral because two enantiomers coexist on a surface after adsorption. Interconversion of such isomers is not possible as it requires flipping the molecule out of plane by 180° in spite of the stabilization of the coplanar configuration by π-metal interactions. Note that contrary to the predominant case of prochiral compounds reported in the previous literature,^10,15,17,18^ the prochirality of TT is not caused by conformational adaptations, e.g., molecular flexure and/or rotations around chemical bonds,^19^ but rather is an inherent property of the molecular structure itself. In addition, the molecule breaks the symmetry of hexagonal or square symmetric substrates and is therefore likely to have a low activation energy for lateral diffusive displacement associated with considerable adsorption energies due to its planarity. This is particularly true for coin metal surfaces, where the adsorption energetics are dominated by the free-electron-like behavior from delocalized electronic states rather than directional bonds.

For all the characteristics listed here, cyclic triimidazoles are very interesting because of their threefold rotational symmetry along the out-of-plane axis. Their lower symmetry weakens the overlap/corrugation of the effective surface potential they experience on substrates with a hexagonal close-packed structure. The balance of very small energy differences (close to degeneracy) is expected to discriminate the *S* and *R* enantiomers on the surface in different physical or chemical processes.

**Figure 1:**
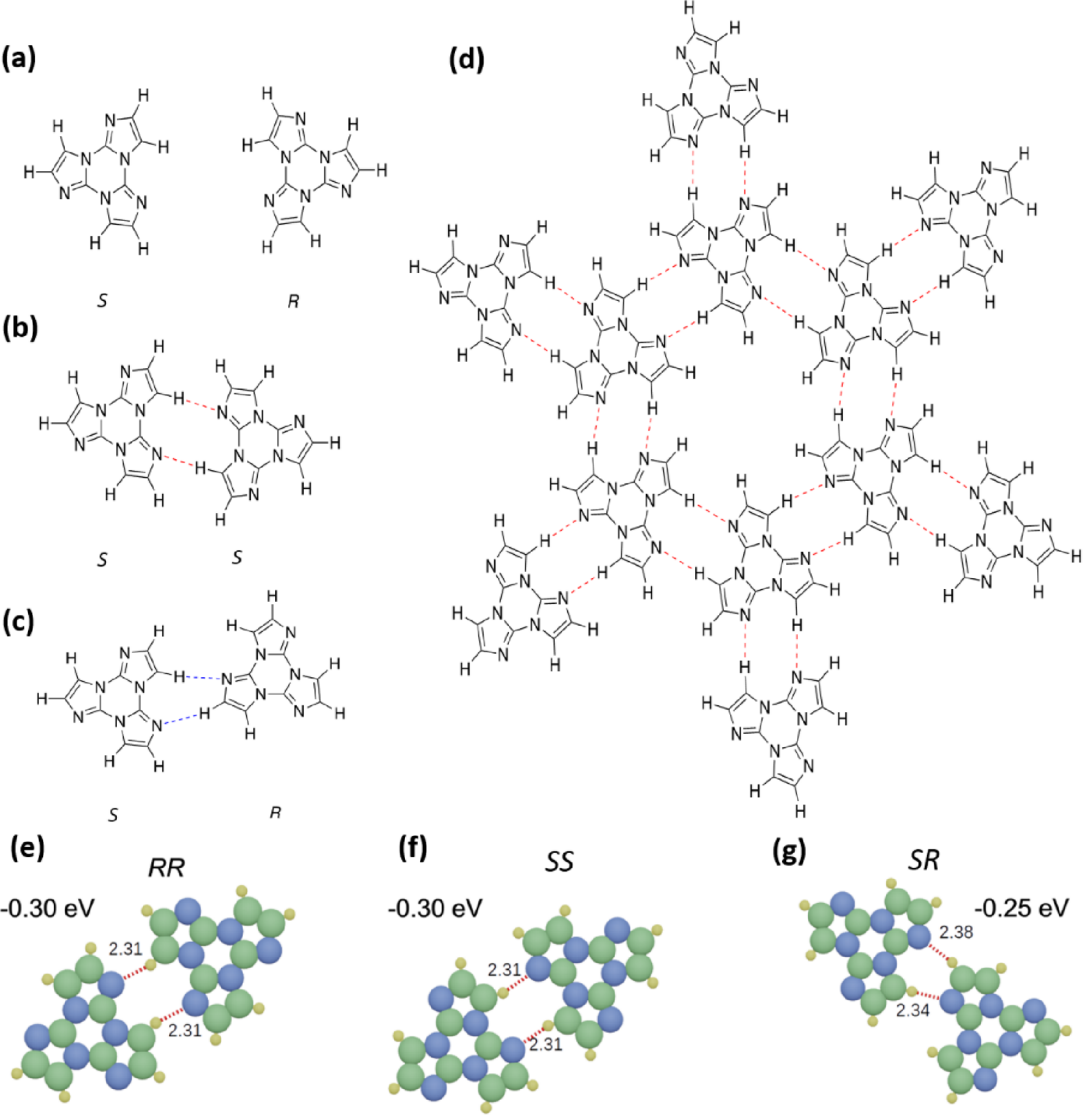
Binding motifs between different pairings of TT surface enantiomers showing mirror images of *S*- and *R*-configurations (a) on the substrate surface. (b) Two *S*-enantiomers of TT adsorbed on the surface form two complementary and symmetrical C–H···N hydrogen bonds. The analogous applies to the two *R*-enantiomers. (c) *SR* dimers can also form extensive periodic assemblies due to two asymmetrical C–H···N hydrogen bonds (see discussion and figures in SI for further information). (d) A fragment of a model for the energetically most favorable (vide infra) homochiral 2D assembly of TT (the *S*-enantiomers are shown). (e–g) DFT computed energies indicating the relative stability of *RR*, *SS,* and *RS* TT dimers relative to two isolated TT molecules (Δ*E* = *E*_dimer_ – *E*_monomer 1_ – *E*_momomer 2_). Hydrogen bonds are indicated by red dotted lines, with their lengths given in Å. C, N, and H atoms are shown as green, blue, and small yellow spheres, respectively.

De Kimpe et al.^20^ reported first steps in the preparation of triimidazo[1,3,5]triazine derivatives with the aim of combining three imidazole moieties in a polycyclic structure. More recently, a synthesis protocol in conjunction with single crystal structure determination of the basic cyclic triimidazole (triimidazo[1,2-*a*:1′,2′-*c*:1″,2″-*e*][1,3,5]triazine, hereafter referred to as TT) was described by Schubert et al.^21^ The molecular structure of the planar polycyclic ring system of TT is shown in Figure 1a.

In recent years, Cariati et al. have extensively studied the class of cyclic triimidazoles and their derivatives for their solid-state luminescent behavior^22,23^ and, in particular, for their crystallization-induced emissive properties.^24−27^ It is noteworthy that TT exhibits electronic properties more likely to originate from three independent imidazole rings, and accordingly, the aromatic sextets are embodied in the imidazole rings. From this perspective, a parallel can be drawn with the extensive research on polycyclic benzotrithiophene compounds.^28,29^ Analogous to TT, the symmetric *D*_3h_ isomer consists of three thiophene units that are electronically only weakly coupled.^30^ These results contrast to some extent with analogous threefold symmetric molecules possessing three peripheral redox centers fused to a central π-system of coronene or hexaazatriphenylene type, in which substantial intramolecular electronic interactions between the redox centers have been detected electrochemically.^31,32^

We provide here the first in-depth analysis of the on-surface energetics of the TT molecule and its enantiomers because this is an interesting case by itself, vide infra, and also because beyond driving the self-assembly, these energetics are crucial for this molecule’s on-surface vs in-fluid reactivity and the reaction energetics and kinetics. The properties of TT molecules on the surface of an Ag(111) substrate and their self-assembly patterns are investigated using scanning tunneling microscopy (STM) and low energy electron diffraction (LEED) experiments in combination with DFT calculations.

## 2 Experimental Methods and Materials

TT was prepared according to the procedure described in the literature^21^ and further purified by repeated crystallizations. The Ag(111) substrate was prepared by cycles of Ar^+^ sputtering at *E* = 1000 eV followed by annealing at 800 K. The TT molecules were deposited by sublimation from a glass crucible kept at room temperature. Exposure was controlled by opening/closing a shutter for an appropriate time, and coverage was estimated by a quartz crystal microbalance and measured by analyzing large-scale STM.

### 2.1 STM Measurements

For STM measurements, bias voltage is applied to the tip, and measurements were performed in constant current mode using Pt–Ir tips (90% Pt, 10% Ir) prepared by mechanical cutting followed by sputtering and STM piezo motion-controlled indentation into the bare Ag(111) substrate. The STM data were analyzed using the WSxM software. Note that all STM data were acquired at 4.2 K

### 2.2 Low Energy Electron Diffraction (LEED) Measurements

The LEED measurements were performed using a V.G. Electrovac LTD 474 unit. The LEED data were recorded at energies between 8 and 68 eV for TT molecules on Ag(111) held at room temperature, whereas LEED data of the clean Ag(111) substrate were obtained at voltages up to 200 eV. The LEEDpat4.3^33^ software was used to simulate the experimentally obtained patterns.

## 3 Theoretical Calculations

Density functional theory (DFT) calculations were performed with the Quickstep code^34^ within the CP2K package using a mixed Gaussian and plane waves basis set; the Goedecker, Teter, and Hutter (GTH) pseudopotentials^35^; and a GGA-PBE^36^ exchange-correlation functional including self-consistently the van der Waals (vdW) interaction.^37^ We used a plane-wave basis-set energy cutoff of 500 Ry and the Γ point to sample the Brillouin zone. The Ag(111) substrate was modeled using a periodically repeated slab of four layers with a vacuum gap of ∼10 Å between the adsorbed molecule and the bottom layer of the slab above it. Relaxations were considered completed when atomic forces converged below 0.02 eV/Å. Computed STM images were obtained by calculating the integrated local density of states (ILDOS) within the Tersoff–Hamann method^38^ using CP2K. The constant current STM images were simulated using the ASE package.^39^

## 4 Results and Discussion

Cyclic triimidazole molecules with their 18 π-electrons are characterized by a compact and rather planar structure exhibiting a threefold rotation symmetry (Figure 1a). *Ab initio* gas-phase molecular orbital calculations of TT in its *C*_3h_ ground-state symmetry were performed at the DFT-PBE level of theory. In agreement with the threefold symmetry, the HOMO is a degenerate pair of π-orbitals with e” irreducible representation located at an energy of −5.71 eV, and the HOMO-1 is a symmetric a” combination of three imidazole MOs located at −6.18 eV, close to the HOMO. The LUMO is analogously a degenerate e” pair of π-orbitals located at −1.59 eV, and the LUMO+1 with its a” representation is located at −0.28 eV, leading to a HOMO–LUMO gap of 4.12 eV. The shapes of the frontier MOs are shown in Figure S1. We also note that three pyrrole-type nitrogen atoms and three peripheral pyridine-type nitrogen atoms, potentially acting as hydrogen-bond acceptors, are characteristic features of the molecular framework. As soon as the rotational vector is fixed in its orientation with regard to the surface plane, two on-surface enantiomers are formed, one left-turning (*S*) and one right-turning (*R*), as shown in Figure 1a. Importantly, two *S*-enantiomers or, similarly, two *R*-enantiomers can pair, respectively, forming two complementary C–H···N hydrogen bonds in a symmetric manner (Figure 1b), whereas the asymmetric hydrogen-bond formation between two different enantiomers (Figure 1c) yields a distinctly reduced binding energy (vide infra). Because of the *C*_3_ symmetry, each enantiomer can generate this symmetric hydrogen-bond pairing in all three directions, so it is straightforward to model the arrangement of the same enantiomers in a two-dimensional assembly. The result is a hexagonal pattern consisting exclusively of enantiomers (*S*- or *R*-enantiomers) and exhibiting organizational chirality (Figure 1d). Triggered by this hydrogen-bonding motif, a fully extended homochiral surface structure with a two-dimensional space group symmetry of *P*6 is formed. We also note that, principally, a pair of *S*- and *R*-enantiomers of TT can form two asymmetrical C–H···N hydrogen bonds (Figure 1c), and such a pairing feature could lead as well to an extended and ordered 2D assembly, however of heterochiral character (Figure S2).

It is widely recognized that the physisorption process for generating surface patterns is highly dependent on the molecular structure, and there is a strong interest in predicting 2D structures based on targeted molecular functionality.^40^ A large number of (sometimes weaker) bonds between different molecular modules are often the origin of complex structure formation and function. This is well-established in molecular life sciences, e.g., protein structures, and in biomolecular recognition processes. Cooperative recognition of multiple supramolecular interactions is also the basis for structural identity, stability, and self-repair. In this regard, a formalism to rationalize network topologies for 3D and 2D crystalline bulk systems formed from weak interactions, e.g., H-bonded in this case, is desired as a unifying basis.^41,42^ In this sense, a chemical entity with three intermolecular binding sites such as TT is described as a three-connected point. A bound pair of these consequently provides four remaining binding sites, and this is precisely the building block required to extend any system into two dimensions, i.e., to have two connecting axes. Consequently, these building blocks are continuously interconnected, and an extended hexameric layer arrangement inevitably emerges as the only possible topological structure (schematically shown in Figure S3). In the actual case, the specific placement of hydrogen-bonding functions on each TT molecule generates the three-armed nuclei and thus the three-connected points that lead to the modeled hexameric patterns (Figures 1d and 4a,b and also Figures S2 and S3). In TT, the chirality aspect does not *a priori* affect the above connectivity rules as long as the molecule acts as a three-connected point, and therefore, either homochiral or heterochiral hexameric 2D patterns are in principle possible. Regular arrangements of sorted *SS* and *RR* dimers were also found in on-surface supramolecular assemblies formed from prochiral porphyrins with phenylene-acetylene functionalization.^19^ Other notable examples confirm this concept as sound, such as the finding that threefold symmetrical benzotri(7-azaindole) molecules deposited on an Au(111) substrate show spontaneous resolution of surface enantiomers forming a hexameric arrangement.^43^ Similarly, spontaneous chiral resolution into ordered honeycomb-like structures was demonstrated in a self-assembly of threefold symmetrical and prochiral tris(2-pyridyl)-triazine molecules on Au(111) by Hou et al.^44^

The chirality and spontaneous resolution of prochiral^10,12−15,17,18,45−56^ and chiral^5,16,57^ building blocks into supramolecular on-surface structures have been discussed in considerable depth, albeit there are still challenges to understanding and controlling chirality on the level of functional groups and interaction forces.^7^ Essentially, there are two basic cases to be distinguished for the type of intermolecular interactions: (i) the weak/less-directional interaction regime and (ii) the opposite case of stronger/more directional interactions. An example for the first case are unsubstituted helicenes, which are well-established in the early work of Ernst et al.^14^ and other authors. An example for the second case is functionalized helicenes with hydrogen bond donor/acceptors, where the considerable strength/directionality of the bond can be modified by metal coordination leading to chain formation, which in some cases promotes enantioselectivity. The role of complementary hydrogen bonds,^58−60^ their C–H···F analogues,^61−63^ and cooperative C–H···N hydrogen bonding on the ordered self-assembly pattern on Ag(111) or Au(111) and their influence on the formation of enantiopure/defective or racemic supramolecular aggregates have been studied with heterocyclic tetraazaperylene, hexaazatriphenylene, or pyrazinophenanthroline molecules.^64−67^ From a kinetic point of view, the formation of achiral structures is facilitated by *SR* pairing as in ref (13), especially when nucleation and growth occur far from thermodynamic equilibrium, because the conformers do not need to separate into homochiral domains.

On the basis of geometric/topological considerations, there are two types of C–H···N hydrogen-bonding patterns between adjacent TT molecules (Figure 1). For free-standing molecules (neglecting surface effects), DFT calculations for symmetric double hydrogen bonds between the same enantiomers yield an energy 0.05 eV (4.82 kJ/mol) lower than for the two asymmetric hydrogen bonds between different enantiomers, which is about twice the thermal energy of 2.5 kJ/mol at room temperature. We note that, against Wallach’s rule,^68−70^ the enantiopure monolayer of TT on Ag(111) (unlike larger Co-diphenylporphyrins on Cu(111)^71^ has a higher density than the heterochiral one (see SI section S7). The calculated (C−)H···N distances, i.e., 2.31 and 2.34, 2.38 Å for homochiral and heterochiral pairs, respectively, as shown in Figure 1e–g, are shorter than those experimentally determined for a large number of bulk crystals, ranging from 2.52 to 2.72 Å.^72^ However, a particularly interesting comparison arises from an experimental and computational study of s-triazine physisorbed on a graphite surface.^73^ The 1,3,5-triazine molecules assemble into a crystalline monolayer that has six C–H···N hydrogen bonds per molecule, and the experimental (C−)H···N distance is 2.383(3) Å. A simulation of the 2D lattice structure on graphite yields intermolecular (C−)H···N distances between 2.31 and 2.39 Å depending on the semiempirical corrections applied. The strength of the intermolecular interactions is estimated to be between 0.09 and 0.14 eV per hydrogen bond depending on the corrections considered. These values agree well with the binding energies of 0.15 and 0.125 eV per hydrogen bond calculated for the homochiral and heterochiral TT dimers, respectively.

**Figure 2:**
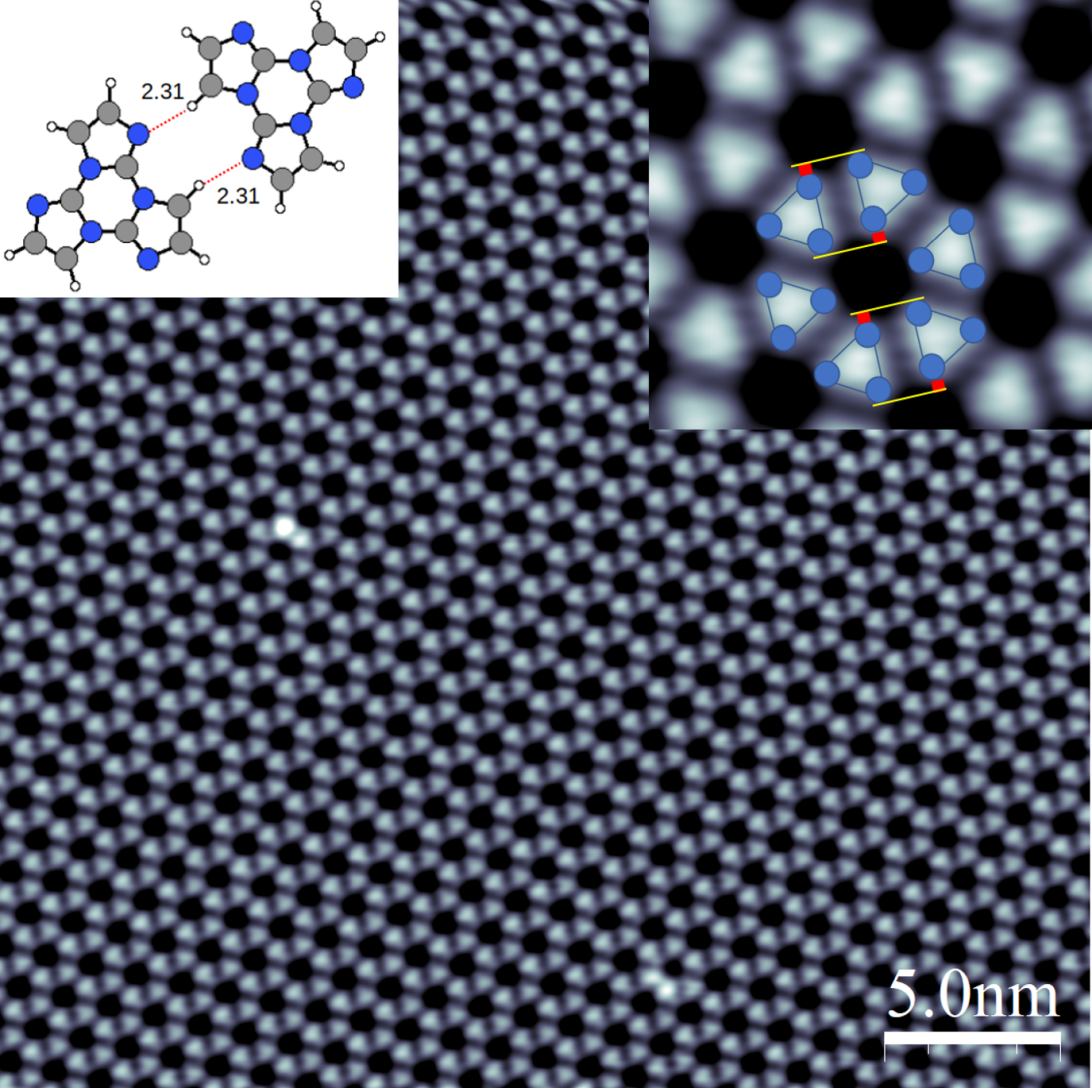
High-resolution STM image (30 × 30 nm) of a monolayer of TT molecules on Ag(111) (*V*_b_ = 1000 mV and *I*_T_ = 40 pA); the inset indicates a *homochiral* domain. This is best recognized by looking at one specific TT dimer in the network. All triangular components are shifted slightly along their contact line as indicated by the red and yellow lines (see Figure S4 for details). Depending on the direction of the shift, two mirrored configurations can be recognized.

To identify the ordering and enantiomeric sorting of TT molecules, STM experiments were performed at submonolayer and nearly complete layer coverage on an Ag(111) single crystal after it had been atomically precisely cleaned. The STM data (Figure 2) reveal that TT self-assembles in an extended hexagonal porous network on Ag(111). Such a network morphology can, in principle, be formed after spontaneous resolution on the surface in two mirror islands by *RR* and *SS* pairing or alternatively in a single heterochiral domain by *RS* pairing. The chirality is best visualized by contact lines parallel to the high-symmetry directions that show a systematic shift between adjacent TT molecules (see Figure S4 for details).

**Figure 3:**
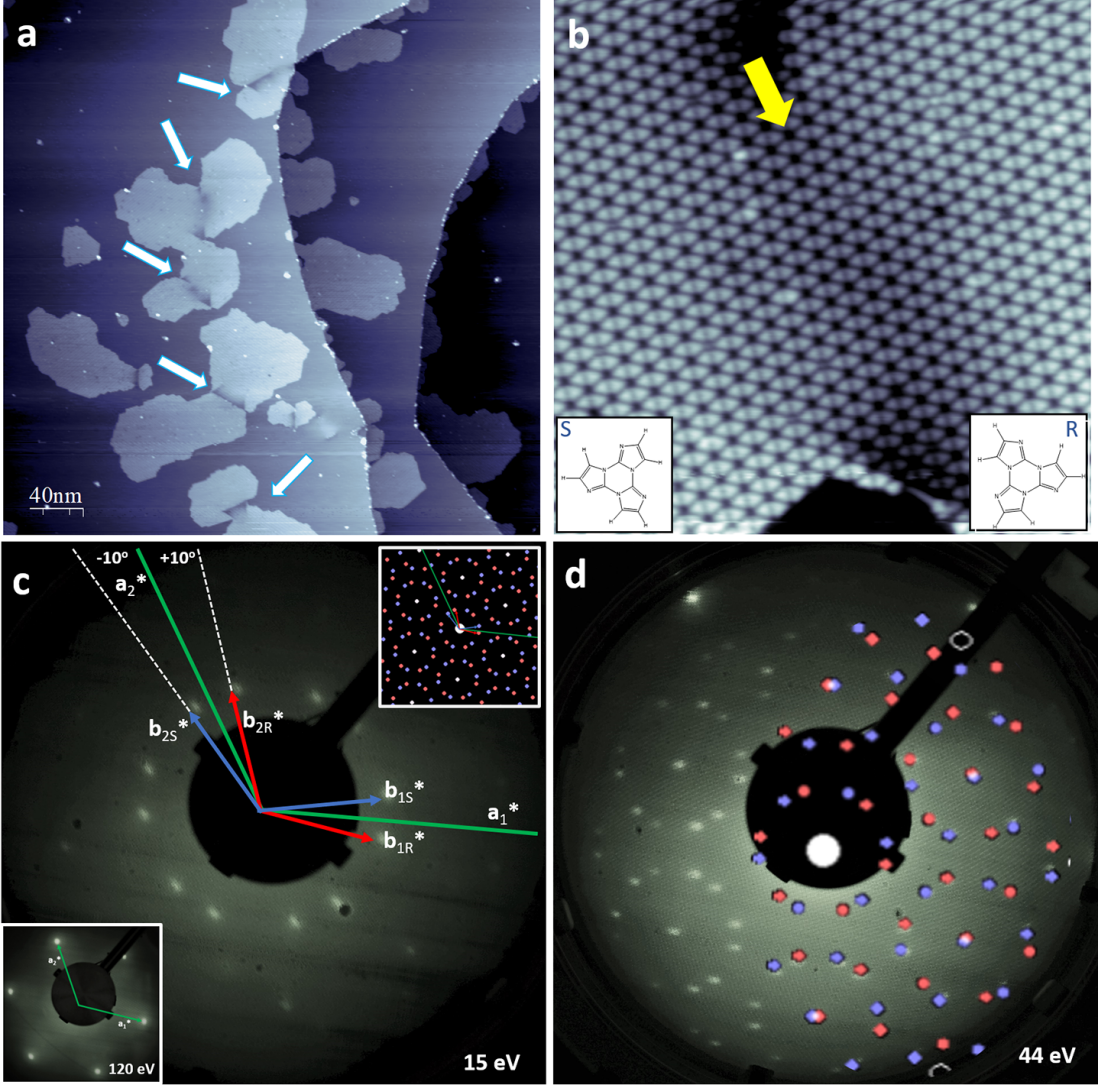
Extended nanoporous 2D networks formed by deposition of TT molecules on Ag(111). (a) STM overview image (400 × 400 nm, 1 V, 20 pA) of the kidney-shaped islands formed by TT. (b) One specific interface zone between two different domains of the hexagonal network. There is a contrast change across the crystallographically “defective” interface zone that we tentatively assign to lattice misfit and density of state (DOS) contrasts (see also discussion in Figure S4) (STM image 40 × 40 nm, 1 V, 40 pA). (c) LEED data of the porous network of TT on Ag(111) taken at room temperature (RT). The diffraction pattern of the porous network of TT was recorded at a beam energy of 15 eV, and that of the Ag(111) substrate was recorded at 120 eV (lower inset). The reciprocal unit cell vectors b_1S_* (b_1R_*) and b_2S_* (b_2S_*) of “*S*” (“*R*”) domains are indicated by violet (coral) arrows. The reciprocal unit cell vectors a_1_* and a_2_* of the Ag(111) substrate are indicated by green lines. The sets of spots for the “*S*” or “*R*” domains draw an angle of ±10° with the principal axes of Ag(111). The simulated LEED pattern is shown in the upper inset of panel c. The experimental LEED pattern of TT, here taken at a higher incident beam energy to show a larger section of the reciprocal space, is characteristic for a *R*19.1° reconstruction, as evidenced by (d) the overlay of the simulated LEED pattern with the experimental data showing a close to perfect match (see also Figure S6 where a LEED pattern with simultaneously appearing substrate and superstructure spots has been analyzed).

To further establish the network formation and symmetry, we performed low energy electron diffraction (LEED) experiments and found evidence for a complex and quite rare surface reconstruction, i.e., the *R*19.1° overlayer structure.^74^ The LEED data are shown in Figure 3c,d and also in Figure S5 together with the corresponding LEEDpat simulations. The match of the experimental and simulated LEED data (LEEDPat4.2 diffraction simulation program^33^) is close to perfect based on the assigned *R*19.1° overlayer on the Ag(111) lattice.

Small adsorbates like Ar and CO on metals, i.e., for adsorbates with simple point/axial symmetry, have been reported to assume a *R*19.1° reconstruction with a smaller than the presently observed unit cell.^75−77^ Note that Baddeley and Richardson^78^ explain two possible causes of chirality in surface reconstructions: (i) symmetry reduction due to the intermolecular bonding pattern (as discussed above) and (ii) the mathematical fact that hexagonal point lattices on hexagonal substrates can form two chiral domains if their rotation is not in sync with the 120° symmetry of the substrate. The *R*19.1° superstructure provides such a case. In the present work, LEED and high-resolution STM images performed in combination for different preparations of TT-covered substrates reveal that the two on-surface enantiomers separate into homochiral “*S*” or “*R*” domains. The chirality of the domains can be recognized by looking at the individual dimers: The molecules with their equilateral shape are slightly shifted along their connection line, as marked in Figure 2 and Figure S4. The 2D networks of TT on the surface consist of regular chiral pores enclosed by six TT molecules. Note that STM and LEED have characteristically different sensitivity to such domains and patterns. Across the spot of the incident electron beam, the LEED pattern is the incoherent superposition of coherently formed diffraction patterns within a coherence length of about 100A. It is therefore very sensitive to the lattice parameters; STM is locally sensitive and can discriminate the domains with lower accuracy distance measurements. The STM contrast results from the different electron densities of the N atoms over the C atoms in the molecular structure. Therefore, STM identifies the local arrangement at the level of a single molecule or single intermolecular bond pair and reveals the chirality of supramolecular islands on a local scale. Further evidence for chirality comes from the remarkable observation of kidney-shaped supramolecular islands that exhibit a narrow connection line that appears to be structurally distorted, such as those shown in Figure 3a and also in Figure S4. In these structures, neighboring domains are frequently connected by a defect zone, the darker appearance of which we associate with a lower density of states available for tunneling STM contrast rather than topography changes. The latter can be excluded as DFT calculations show negligible variations in height above the surface for different binding sites (Figure S8a,b). We take these, in some cases sharply delineated, zones as the counterface of a domain boundary, and their rather short length compared to the circumference of the kidney-shaped islands indicates that their growth is retarded/energetically unfavorable compared to the extension of the neighboring islands. Note that the domain boundary is difficult to be recognized possibly because of the small triangular shape of the molecular module and the rather large superstructure. This is in spite of the structure needing to accommodate for some of the crystallographically possible misfits/misorientations between neighboring islands as well as eventually for the chirality mismatch. Note that we have also considered the hypothesis that the domain boundary is created by a vacancy island in the substrate due to the inclusion of Ag atoms in the network formation. We take the absence of changes in the island morphology and its fine structure (Figure S9) as evidence against such a mechanism. The frequency of their occurrence, however, suggests that the binding or 2D condensation energetics of the domain boundary remains positive (over the formation of separate islands) despite the compromised intermolecular binding and substrate registry. We may tentatively propose that such “kidneys” occur by nucleation, already at an early stage of growth, of *S* enantiomers to *RR* islands (and vice versa), as kidneys are observed already at very early stages of growth. This may occur by capturing any ad-molecules during the deposition process. Whereas *R* conformers will aggregate to the capturing *RR* islands, *S* conformers aggregating with *RR* domains diffuse along the island boundary to the neighboring *SS* domains and vice versa. Another possibility would be a ripening and expulsion process starting from heterochiral nuclei. Additional data are shown in Figure S4. We note that it is impossible to state, based on our data, if the walls are molecularly sharp, optimizing intermolecular interactions over strain, or diffuse, minimizing strain at the expense of sub-optimal hydrogen bonding.

**Figure 4:**
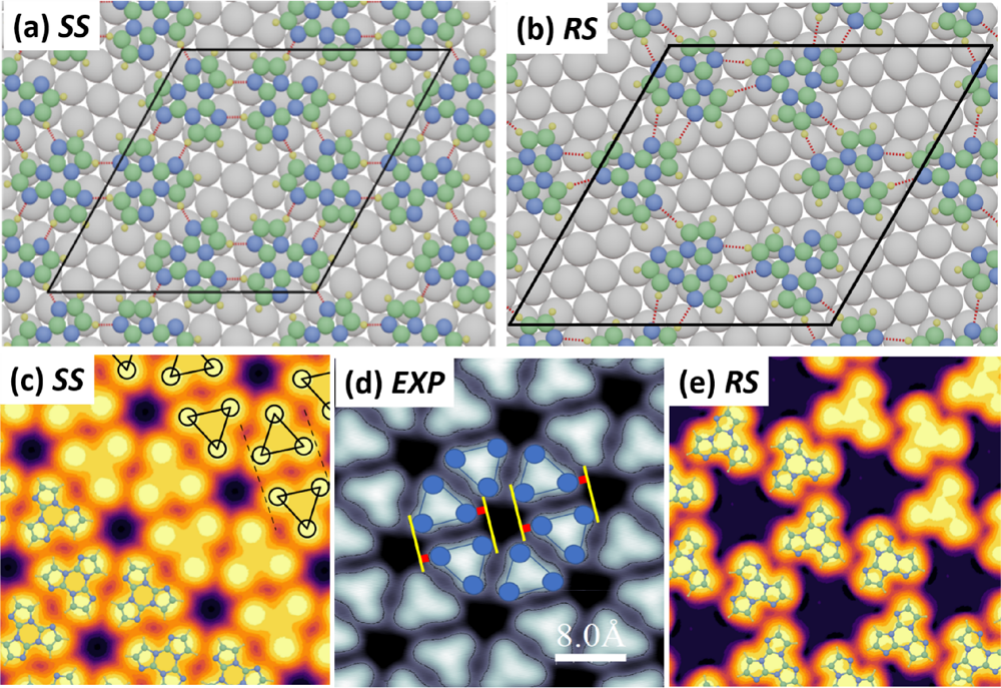
Most stable *SS* (a) and *RS* (b) configurations for a TT monolayer adsorbed on the Ag(111) surface. Note that the graphs in each row are drawn to scale; i.e., the unit cell of the heterochiral *RS* assembly is larger than the homochiral *RR* or *SS* assembly. Simulated STM images for *SS* (c) and *RS* (e) monolayers compared to the experimental STM image of a *SS* region (d) (size STM image 4 × 4 nm, *V*_b_ = 1 V, and *I*_T_ = 40 pA). The dashed lines plotted in panel c clearly delineate a parallelogram evidencing that there is a shift—and therefore chirality—observed in the DFT optimized structure. In panel d, the lateral shift of the triangular molecules has been estimated (red bars ∼1/2 Å) by adding yellow lines that are perpendicular to the basis of the triangular units forming one dimer in the superstructure.

To obtain an atomistic understanding of the structures of the TT monolayers, we carried out DFT calculations for different molecular arrangements based on the experimental information obtained from the STM images and LEED. The dimers shown in Figure 1 were placed within a hexagonal unit cell (Figure S7), and the stability clearly shows the same trend as for the isolated dimers, with *RR*/*SS* dimers being more stable than *RS* dimers by 0.08 eV. In addition, the interaction between a single TT molecule and the Ag(111) surface was studied, and positioning of the TT center at the on-top site of the silver surface was found to be 0.06 eV more stable than at the second-most favorable hollow sites (Figure S8). Homo- and heterochiral TT monolayers lying on top of a silver slab were then calculated (Figure 4 and Table S1). The *RR*/*SS* networks were distinctly more stable compared to the *RS* one, while also all central aromatic rings of TT are located at on-top sites of the silver surface. The simulated STM images of the *SS* and *RS* configurations are shown in Figure 4.

This DFT data can also be used to approximate the energy of the defective zone between two enantiomeric islands. If we consider that per TT molecule the hydrogen-bond energy is reduced by 0.08 eV for the necessary *RS* contact and, in addition, the potential energy is raised by a maximum 0.06 eV when the molecule is shifted away from the on-top site, an energy cost of maximally 0.14 eV per TT molecule arises in the domain-wall defect zone.

The present analysis sheds some light on the remarkable characteristics of the supramolecular self-assembly of these triimidazoles. First, the energy difference between *SS*, *RR,* and *RS* patterns is such that spontaneous resolution occurs, which is often desired but not always observed. The vast majority of TT molecules is found in homochiral 2D islands. Second, the difference in the binding scheme or binding energy between *RS* bonds is not yet significant enough to completely separate *RR* and *SS* islands. Surprisingly, domains of TT are often connected. This is not typically observed in conjunction with spontaneous resolution. Third, we show that “faulty” contacts occur regularly in the form of domain boundaries, indicating a low barrier to molecular displacements from the energy minimal epitaxial situation, possibly in the order of fractions of the substrate lattice constant. This observation agrees well with the DFT result that the binding energy of *RS* islands is energetically close to the one within homochiral *RR* and *SS* islands. Note that there is a plethora of different possible domain boundary morphologies because the many possible forms of mismatch between the *RR* and *SS* domains lead to a multitude of different boundary conditions. Figure S6 shows a selection of experimentally observed domain boundaries. It is important to note that these many morphologies at the domain boundary are also a consequence of the “elasticity” of H-bonding: neither the bond distance nor the exact angular arrangement is strict, allowing for a multitude of arrangements that cause the variability of the system. Note that the 2D islands also like to nucleate on the lower terrace next to single steps of the Ag(111) substrate by bonding or coordination after reactive desorption of H atoms. Spontaneous resolution, as has also been reported for a similar molecule,^43^ is plausible for the formation of Janus-shaped island pairs, but does not lead to them. In our system, there is a distinct supramolecular recognition motif involved in the formation of the layer but at the same time appears to be the cause of the segregation/sorting of the enantiomers. This recalls the importance of interadsorbate distance requirements and the registry with the substrate. Our results show that supramolecular control depends on the complexity of competing interactions and is not possible in the case of centrosymmetric pair potential interactions.

## 5 Conclusions

We have studied the self-assembly of pro-chiral cyclic triimidazoles on Ag(111) and observed spontaneous resolution. We find evidence for “Janus-pairs” of islands that we attribute to interenantiomer condensation occurring between homochiral islands. The system is hence considered a “limiting case” where neighboring phases are connected by a narrow, crystallographically “defective” zone of molecules on the hexagonal Ag(111) substrate. In this characteristic fault zone, the benefits due to interisland bonding are balanced at the expense of lattice mismatches and strain. We associate a considerable but weakly directional bonding scheme as it clearly goes beyond vdW condensation due to the three N-containing five-membered imidazole rings.

DFT calculations show that for both free-standing dimers and monolayers and for surface-confined monolayers, the homochiral pairwise interactions are energetically more stable than the heterochiral ones. Importantly, the studies of the supramolecular assembly of racemates equipped with different symmetry and different directional binding motifs provide the basis for further studies to control spontaneous resolution in on-surface assemblies and reactions. This is an important key for further advances in on-surface synthesis of, for example, graphene-like islands and ribbons directed by dehydrogenative C–C coupling reactions at higher temperatures.^66,79^ Similarly, a further study will investigate the Ullmann coupling reaction based on suitably substituted dibromo and tribromo TT precursors, which can lead to extended, chiral, and strongly N-doped covalently linked single-layer networks. These intriguing prospects for further research on TT and its derivatives underscore the importance of the present study.

## Supporting Information Available

Frontier molecular orbitals of TT, 2D-self-assembly of TT, a hexameric pattern, STM studies of *S* and *R* enantiomers, a series of LEED patterns of TT deposited on an Ag(111) substrate, and the detailed information on DFT calculations (PDF)
