# Supplementary material for: Self-Assembly of N-Rich Triimidazoles on Ag(111): Mixing the Pleasures and Pains of Epitaxy and Strain
Source: J Phys Chem C Nanomater Interfaces. 2023 Nov 17;127(47):23000–9. doi: 10.1021/acs.jpcc.3c03325 (PMC10694807; doi:10.1021/acs.jpcc.3c03325)
Supplement: Supplementary file 1 — jp3c03325_si_001.pdf [file jp3c03325_si_001.pdf]

Supporting Information

Self-Assembly of N-rich Triimidazoles on Ag(111): Mixing the Pleasures and Pains of Epitaxy and Strain

Aisha Ahsan,[†,⊥] *Xing Wang,*[‡] *Rejaul Sk,*[†,⊥] *Mehdi Heydari,*[†,⊥] *Luiza Buimaga-Iarinca,*[∥] *Christian Wäckerlin,*[⊥] *Elena Lucenti,*[∥] *Silvio Decurtins,*[‡] *Elena Cariati,*[∥,§] *Thomas A. Jung,*[†,⊥,*] *Ulrich Aschauer,*[‡,¤,⊥,*] *and Shi-Xia Liu*[‡,*]

thomas.jung@psi.ch; ulrich.aschauer@plus.ac.at; shi-xia.liu@unibe.ch

Table of Content

1. Molecular orbitals of TT — Figure S1
2. 2D *RS* assembly of TT — Figure S2
3. Hexameric pattern — Figure S3
4. *S* and *R* enantiomers — Figure S4
5. LEED diffraction patterns — Figure S5
6. STM images of kidney structures grown with increasing molecular coverage — Figure S6
7. DFT calculations
   The configurations of the free-standing and adsorbed TT monolayers — Figures S7-S8
   Structure models of Ag(111) supported TT monolayers — Table S1
8. Annealing effect on assembly — Figure S9

1. Molecular orbitals of TT

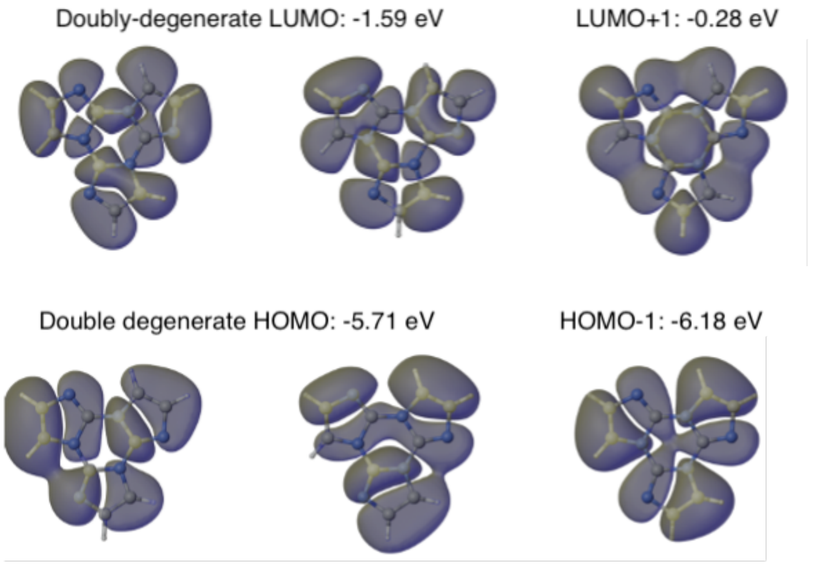

Figure S1. Frontier molecular orbitals of TT with corresponding energies, calculated for a C_{3h} ground-state symmetry.

2. 2D RS assembly of TT

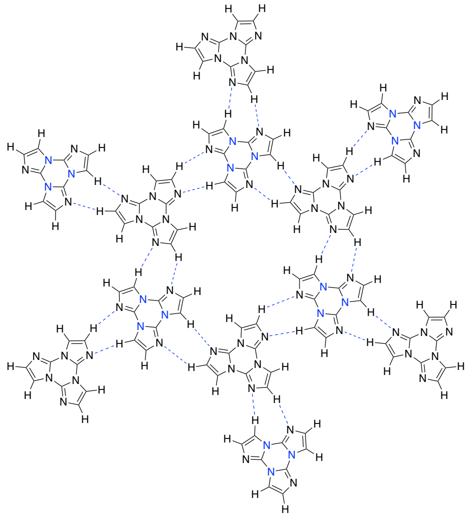

Figure S2. A fragment of a model for the heterochiral 2D assembly of TT based on asymmetrical C–H···N hydrogen bonds between *R*- and *S*-enantiomers.

3. Hexameric Pattern

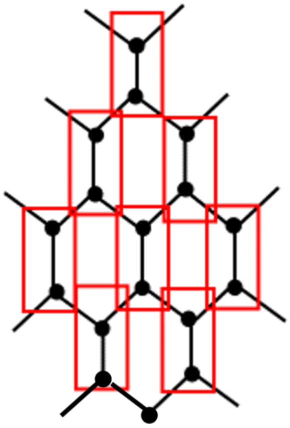

Figure S3. A pair of 3-connected points (inside a red rectangle) has four binding sites, e.g. the four edges of the rectangle. Therefore expansion in two dimensions inevitably leads to a hexameric pattern.

4. S and *R* enantiomers - STM studies

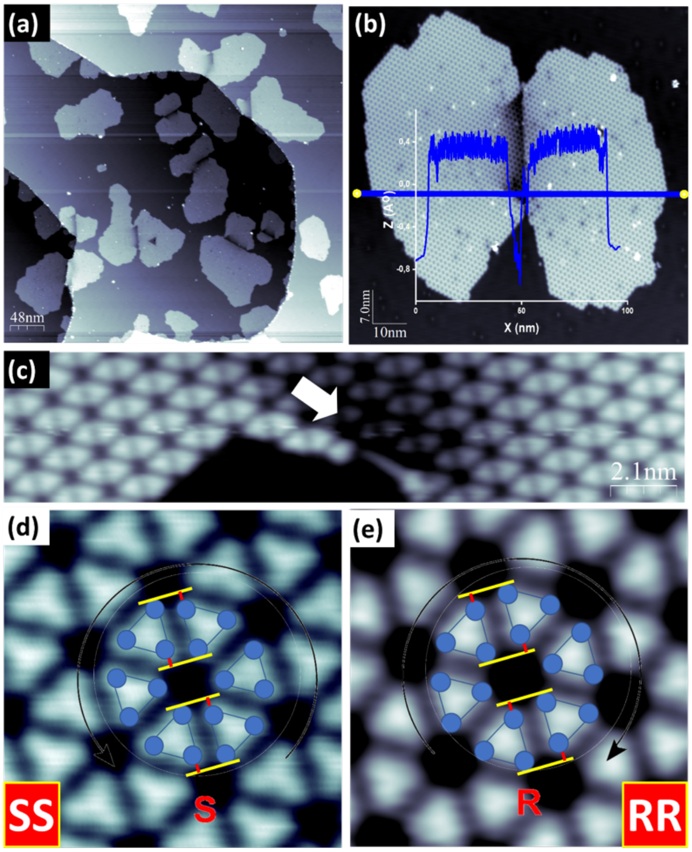

Figure S4. Comparison of two different domains to unambiguously determine the adsorption of TT in enantiomorphic "*S*" and "*R*" domains. (a) Kidney shaped, adsorption of TT molecules on Ag(111) substrate in different domains. (b) A domain boundary (dark region) between domains where a cross-sectional profile analysis has been performed as indicated by the blue line. Remarkably, the magnitude of the apparent height change is in the order of a major fraction of a substrate step height. (c) The domain boundary area has been indicated by a white arrow in a single frame and therefore with the same STM tip. The domain boundary appears in the form of dark arrays of hexagonal pores observed between two supramolecular kidney-shaped islands. (d) and (e) show islands with hexagonal *SS* and *RR* pores, yellow lines are drawn perpendicular to the 'basis' of the triangular molecules within a contacting pair. The red bars indicate the shift, for *S* and *R* in opposite direction to visualize the chirality of the islands.

5. LEED diffraction patterns

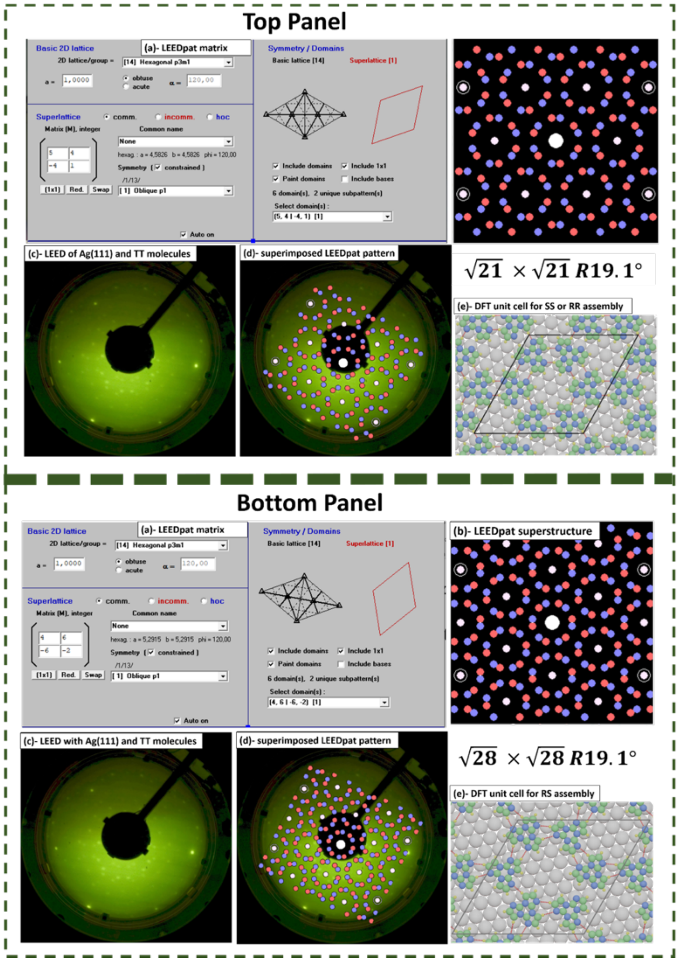

Figure S5. LEED patterns of TT deposited on a Ag(111) substrate are compared to LEED simulations for two superstructures that create similar LEED patterns. Taking the substrate spots as the reference, the measured LEED spots (white spots with circles) correspond only to the $\sqrt{21} \times \sqrt{21}\, R19.1°$ (top panel), not to the $\sqrt{28} \times \sqrt{28}\, R19.1°$ (bottom panel) reconstruction. For the latter reconstruction more superstructure spots can be counted between neighboring pairs of substrate spots. The spots originating from the two chiral domains are shown in different color, white spots correspond to diffraction maxima of both domains.

6. STM images of kidney structures grown with increasing molecular coverage

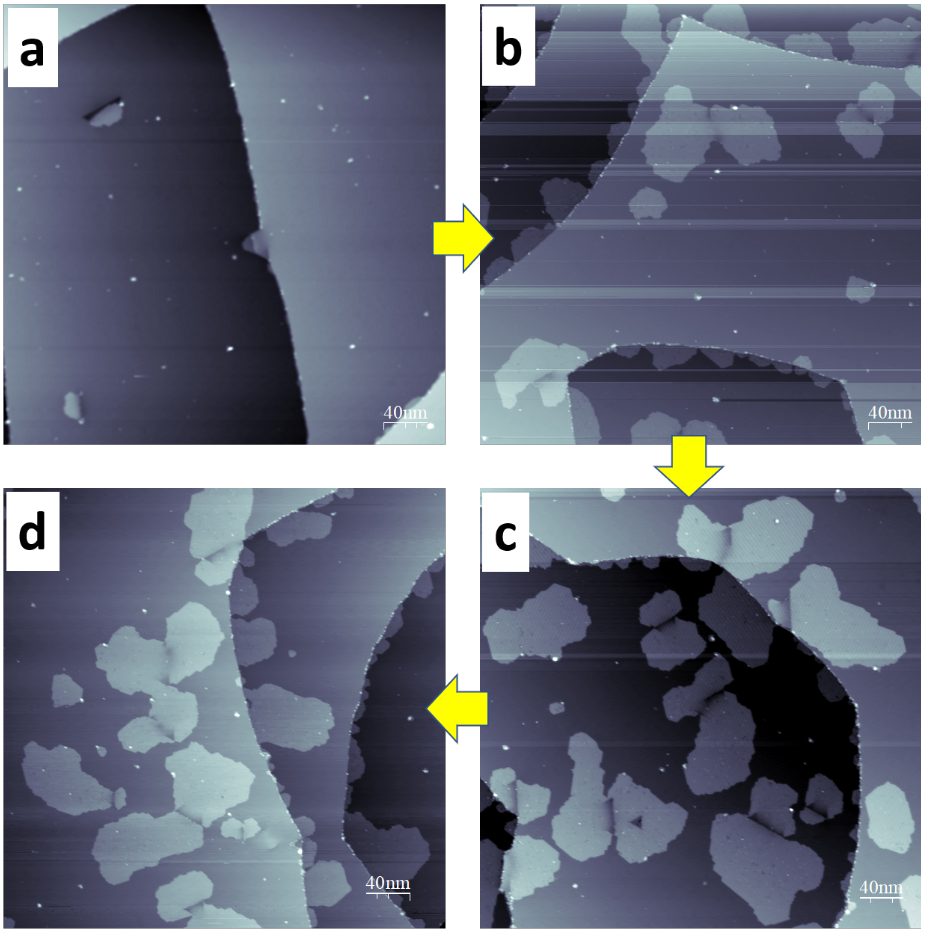

Figure S6. Kidney structures grown with increasing molecular coverage. (a) – (d) from (a) lower coverage to high coverage (d) more of the kidney shaped Janus pairs can be seen. Only very rarely isolated domains are observed, most of those nucleating from a lower step edge. It appears that with progressive deposition the Janus pairs grow by preferential adsorption of S/R modules to S/R domains with a retarded growth of the domain boundary / contact line between the islands.

7. DFT calculations

Calculations of isolated dimers and molecules were performed in unit-cells with 10 Å vacuum along all directions.

RR and RS dimers were placed within a hexagonal unit cell (the same geometry as the Ag(111) surface), forming slabs of free-standing TT monolayers, as shown in Figure **S7**. After geometry optimization, the *RR* (*SS*) monolayers have a lattice constant of 13.47 Å, while the *RS* monolayer has a lattice constant of 14.16 Å. The isolated dimers in the *RR* (*SS*) monolayer are more stable than those in the *RS* monolayer by 0.08 eV.

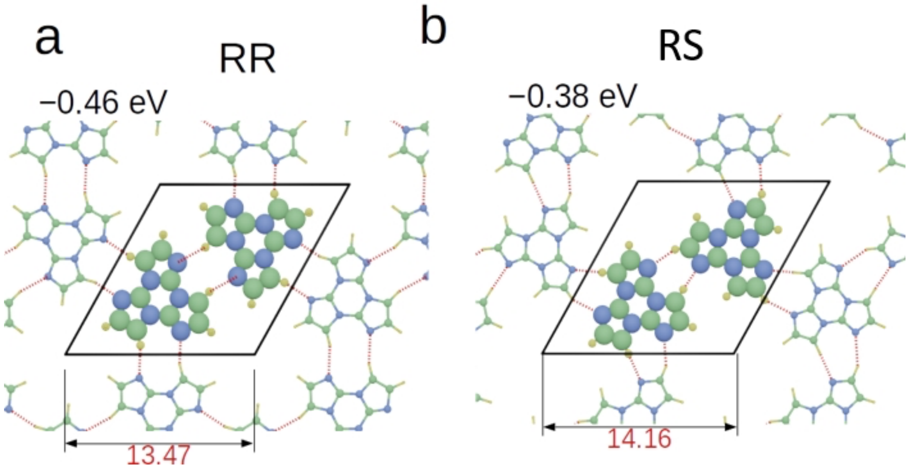

Figure S7. Configurations of the TT monolayers. Energies in eV correspond to the relative stability of each monolayer compared with isolated TT molecules. Hydrogen bonds are indicated by red dashed lines. Dimensions of the hexagonal unit cells are indicated by arrows. C, N and H atoms are shown as green, blue and small yellow spheres, respectively.

Next, we studied the interaction between a single TT molecule and the Ag(111) surface. A TT molecule is placed on a Ag(111) surface with its central aromatic ring on the high symmetry sites (on-top, fcc, hcp and bridge) of the surface. After relaxation, the most stable configuration is obtained if the TT molecule is placed with its center at the on-top site, as shown in Fig. **S8**a. The next meta-stable adsorption sites are the hollow (fcc and hcp) sites as shown in Fig. **S8**b, which are 0.06 eV less stable compared to the on-top site. The bridge site is unstable for TT adsorption, the molecule relaxing to the hollow configuration. The adsorbed molecules remain

in a planar geometry, with a slight buckling of only 0.15 Å. Removing the van der Waals corrections modifies the adsorption height significantly (3.20 Å versus 3.77 Å) as shown in Fig. **S8**, indicating the important role of van der Waals interaction for the silver-TT interaction.

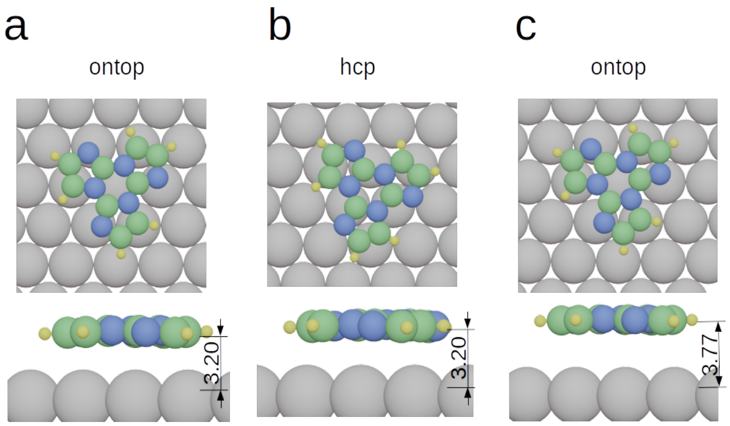

Figure S8. Top and side views of the most stable configuration found for one TT molecule adsorbed on the Ag(111) surface: a) on-top site, b) hcp site, c) on-top site without van der Waals corrections.

Finally TT monolayers were computed on a Ag(111) surface modeled by four atomic layers, with the positions of the two bottom layers fixed at bulk positions. The TT monolayers were deposited on top of this Ag slab. It is assumed that the surface dimensions do not change significantly due to the monolayer, so the lattice constant of bulk silver is used and fixed for all models. Since the primitive monolayer slabs and the Ag surface have different lattice constants, special care is needed in constructing the atomistic models to minimize strain. Table **S1** shows the two models used in our calculations.

Table S1. Structure models of Ag(111) supported TT monolayers along with their energies (in eV) compared to isolated TT molecules.

Model	Ag substrate		Monolayer		Matrix	Relative stability	
	Surface	Supercell	Surface	Supercell		*RR* / *SS*	*RS*
1	$\sqrt{7} \times \sqrt{7}$	2×2	primitive slab	1×1	[(10, -2), (2, 8)]	-1.62	-1.72
2	$\sqrt{7} \times \sqrt{7}$	3×3	$\sqrt{3} \times \sqrt{3}$	1×1	[(6, 3), (-3, 9)]	-1.78	-1.57

The *RS* monolayer is more stable in model 1 due to its larger lattice constant, while *SS* (*RR*) is more stable in model 2. The overlayers have hence been considered within their respectively most stable superstructure, which is given in matrix notation in Table S1. Overall, model 2 with *SS* (*RR*) is the most stable structure. In this structure, different to that of model 1, all central aromatic rings of the TT molecules are located on the on-top sites, which is also the most stable adsorption site for isolated TT molecules. Thus, the *SS* (*RR*) configuration is preferred for the TT monolayer.

8. Annealing effect on assembly

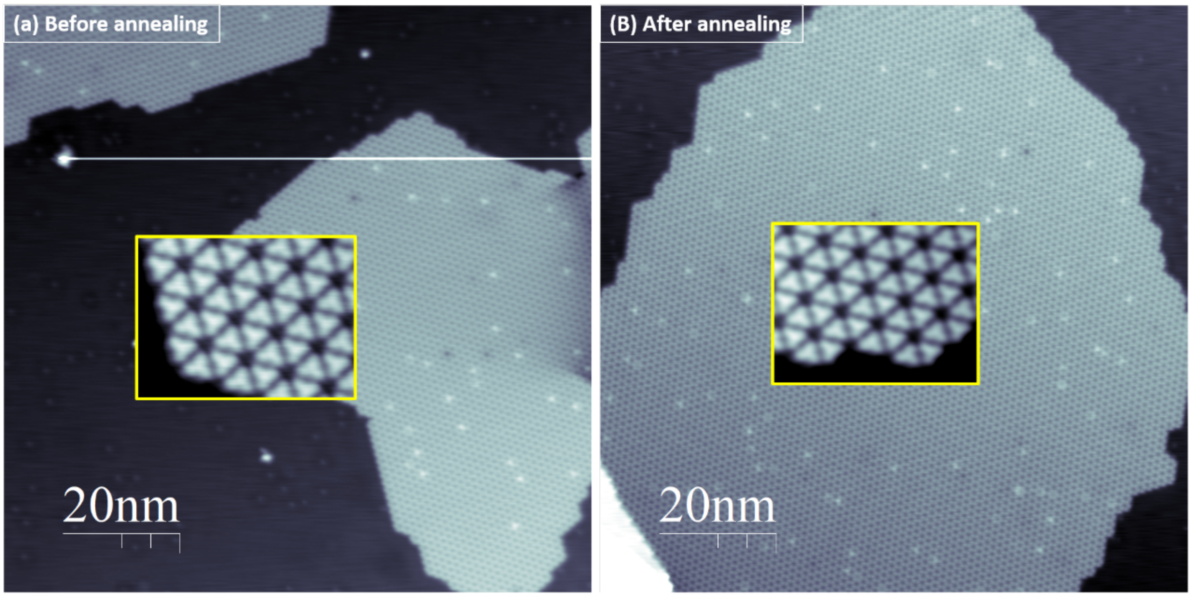

Figure S9. Annealing temperature dependent self-assembly of TT on Ag(111): To rule out the possibility of Ag adatoms linking the TT molecules by their preparation at room temperature samples have been heated up to 125 C. Note that TT Molecules desorb at temperatures above 150 C. We take it as evidence against a significant role of Ag adatoms linking TT in the network formation that neither the network morphology nor its appearance in zoom STM images is changing in an apparent way.
